# PD185 Is The Incorporation Of Medications For Ultrarare Diseases In Brazil Predominantly Driven By Costs? A Shift In Paradigm

**DOI:** 10.1017/S0266462324004070

**Published:** 2025-01-07

**Authors:** Daniel da Silva Pereira Curado, Juliana Machado-Rugolo, Denis Satoshi Komoda, Monica Aparecida de Paula de Sordi, Juliana Tereza Coneglian de Almeida, Alan Fonseca, Silvana Molina Lima, Silke Anna Theresa Weber, Luis Gustavo Modelli de Andrade, Jean-Eric Tarride, Lehana Thabane, Marilia Mastrocolla de Almeida Cardoso, Marisa Santos

## Abstract

**Introduction:**

Incorporating technologies for ultrarare diseases (URD) poses challenges for global health technology assessment (HTA) agencies. Difficulties include defining an analytical framework and establishing differentiated cost-effectiveness thresholds. The rise of technological innovations intensifies demands from healthcare professionals, media, and the general population, placing pressure on healthcare systems in developing countries.

**Methods:**

To analyze ultrarare medicine costs in submissions to the Brazilian National Committee for Health Technology Incorporation (CONITEC), data from HTA reports on URD (from 2012 to 2022) were extracted. Diseases were classified as URD based on an epidemiological criterion or Orphanet consultation (prevalence ≤1 per 50,000 inhabitants). Extracted variables included initial and final prices, annual patient cost, incremental cost-effectiveness ratio (ICER), and initial and final CONITEC recommendations. Price differences were calculated by the Brazilian Medicines Market Regulation Chamber.

**Results:**

Among 53 reports, 30 featured economic evaluations, with only 13.3 percent initially receiving positive recommendations. However, eight gained favor, including post-consultation, price-conditioned, and risk sharing-based approvals. Annual medication costs ranged from USD17,439.20 to USD1,108,237.00 per patient, with discounts of between 25 and 64 percent. Despite some technologies having ICERs that were significantly higher than the national threshold, no statistical relationship was found between ICERs and recommendations. Monthly and annual costs varied, with higher costs for heterogeneous diseases and lower costs for metabolic conditions. Sensitivity analyses, using both deterministic and probabilistic analyses, were conducted in 58 percent of the reports.

**Conclusions:**

Incorporation of technologies for URD does not correlate with lower annual costs or increased discounts because costs are not considered in isolation by CONITEC’s decision-making process. Recognizing URD as a subgroup with distinct criteria may enhance the implementation of HTA processes tailored to the unique challenges of these conditions.

